# Leveraging CNN and Transfer Learning With EfficientNet for Enhanced Optical Coherence Tomography (OCT)

**DOI:** 10.1155/ijbi/1272954

**Published:** 2026-09-07

**Authors:** Siddartha Arekanti, Ch. Madhu Bhushan, Irfan Alam, Priya Koppuravuri, Fazal Shaik, Md Muzakkir Hussain, Firoj Gazi

**Affiliations:** ^1^ Department of Computer Science and Engineering, SRM University-AP, Amaravati, Andhra Pradesh, India; ^2^ Faculty of Computer Studies, Arab Open University Bahrain, A′ali, Bahrain

**Keywords:** EfficientNet, optical coherence tomography (OCT), retinal disease classification, support vector machine (SVM), transfer learning

## Abstract

Optical coherence tomography (OCT) is an important imaging modality for detecting retinal disorders, including diabetic macular edema (DME), choroidal neovascularization (CNV), and drusen, as well as distinguishing healthy retinal patterns. Nevertheless, visual assessment of OCT scans is labor‐intensive, observer‐dependent, and dependent on expert clinical knowledge, encouraging reliable automated screening tools. This work introduces a hybrid Deep Transferred EfficientNet and Support Vector Machine (DTE‐SVM) approach for multiclass OCT image classification. In contrast to standard CNN models that commonly use a softmax layer for final prediction, the proposed framework employs transfer‐learned EfficientNet representations with an SVM decision model to strengthen interclass discrimination and robustness within the extracted feature space. Experiments used a working subset of 8767 OCT images selected from the OCT2017 dataset, representing nearly 10.4% of the complete 84,495‐image collection. This smaller yet representative subset was adopted to lower computational cost while maintaining class distribution among CNV, DME, drusen, and normal groups. Five EfficientNet architectures, from B0 through B4, were assessed using stratified 10‐fold cross‐validation. Among them, DTE‐SVM‐B0 produced the strongest overall results, obtaining an accuracy of 0.9076, precision of 0.9074, recall of 0.9076, and F1‐score of 0.9073. DTE‐SVM‐B2 showed the smallest fold‐level standard deviation, reflecting comparatively stable behavior. Comparative experiments indicated that DTE‐SVM‐B0 surpassed ResNet50, DenseNet‐121, and EfficientNetB0 softmax‐based classifiers, whereas ablation testing verified that substituting the softmax layer with SVM improved results for every EfficientNet backbone. Class‐level clinical evaluation also revealed strong sensitivity and specificity, with CNV identified most accurately and drusen remaining the hardest category. Overall, these results suggest that compact EfficientNet feature extractors integrated with SVM offer accurate, stable, and computationally practical OCT‐driven retinal disease classification for clinical decision‐support settings.

## 1. Introduction

The technique of cross‐sectional imaging of ocular tissue utilizing optical coherence tomography (OCT) has become a widely established, noninvasive retinal imaging technique. Within modern ophthalmology, OCT is extensively used for diagnosing and monitoring numerous retinal diseases, including diabetic macular edema (DME), choroidal neovascularization (CNV), age‐related macular degeneration (AMD), glaucoma, and other abnormalities that may seriously threaten vision [1–4]. By its ability to resolve small microstructural changes within the retina, this modality can be used by clinicians to inspect individual retinal layers, identifies early signs of disease‐related changes, and tracks pathological progression with clinical precision. Since OCT does not involve invasive treatment, it is especially useful in cases of repeated assessment and follow‐up. Nevertheless, with all these advantages, the OCT image analysis remains a complicated clinical task. These pictures are rich in both anatomical and pathological anatomy and the reliable interpretation of such images usually requires knowledge of specialists. In clinical practice, manual examination may be sluggish, subjective, and only apparent at the outset. These issues have led to a rise in the need to have automated diagnostic systems that can assist ophthalmologists in terms of speed, consistency, and reliability when analyzing OCT images.

**Figure 1:**
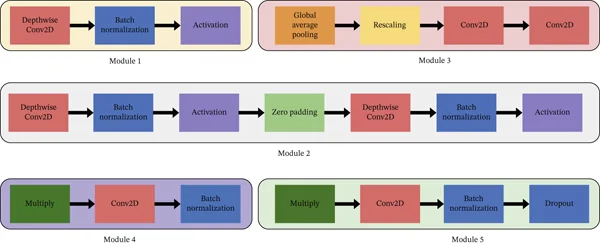
Modules in EfficientNet architecture.

Recent advancements in deep learning (DL) have opened up a host of possibilities to the automatic retinal disease detection problem, and convolutional neural networks (CNNs) have become a very effective solution to the medical image classification problem. Recent CNN architectures such as EfficientNet have received significant research attention because of their ability to offer an effective trade‐off between classification performance and computational complexity of a neural network model, for example EfficientNet ^5,^ [5]. Initially introduced as Google AI (artificial intelligence), EfficientNet uses compound scaling, where the network depth, width, and image resolution are increased in a balanced manner, allowing richer learning of features, but avoiding the excessive expansion of parameters that is inherent to traditional models like ResNet and VGG. This property is very applicable in medical imaging, where practical systems need to have a high level of diagnostic capability as well as realistic implementation. With these advantages in mind, the current study analyzes EfficientNet in addition to a support vector machine (SVM) classifier to classify automated OCT images, specifically in detecting clinically significant conditions of the retina, including CNV, DME, and drusen [6]. Since these diseases have a significant role in visual disability and irreversible loss of vision, early and reliable diagnosis is crucial in timely treatment. This study intends to create a reliable and clinically feasible computer‐aided system in the diagnosis of retinal diseases by incorporating transfer learning, EfficientNet‐based feature extraction, and SVM‐based decision‐making processes only to mention a few features of this study proposal (see Figure 1).

Even though previous studies have been promising in the classification of OCT images, there are some critical issues. Most current methods primarily emphasize predictive accuracy, with relatively little focus on computational efficiency, generalization ability, and preparedness to be integrated into clinical workflows in the real world. In addition, hybrid schemes combining lightweight deep feature learners with strong margin‐based classifiers have not been adequately studied to be applied in the detection of retinal disorders. This weakness constitutes a valuable research gap, especially in the context where the accurate classification is to be obtained within the limited computational resources. The significance of this work is thus based on the suggestion of a diagnostic plan, which takes into account the performance and the practical relevance. The proposed method is expected to support scalable, reliable, and accessible OCT‐based screening both in advanced medical centers and in resource‐constrained healthcare settings. To this end, the work will add to the field of automated retinal disease diagnosis and contribute to the overall goal of creating AI‐assisted ophthalmic solutions that are reliable, deployable, and clinically meaningful in diverse healthcare settings.

### 1.1. AI and DL in OCT

**Figure 2:**
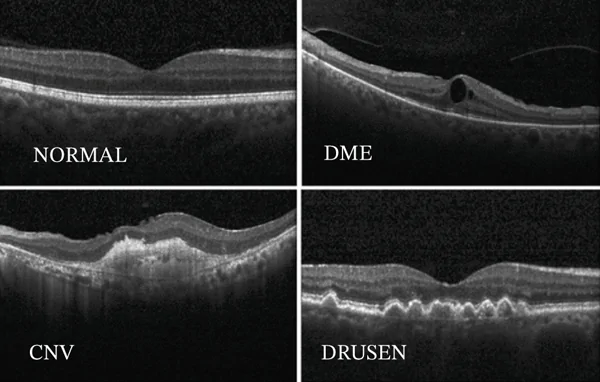
Eye diseases.

OCT has emerged as a powerful diagnostic imaging technique in ophthalmology, offering noninvasive and high‐resolution visualization of retinal structures [7, 8]. It has been widely used in the identification of a number of retinal disorders, including DME, CNV, AMD, glaucoma, and drusen as shown in Figure 2. Unless they are identified and dealt with early enough, they can lead to severe visual impairment or even blindness. However, despite the fact that OCT scans provide great detail about the anatomy, their clinical interpretation continues to be a challenging issue and is often left to skilled experts. The increased need to have accurate, fast, and automated OCT assessment has led to the implementation of AI, particularly DL, in medical image analysis process [9]. The AI‐based DL methods can significantly enhance the interpretation of OCT images, automate disease detection, and classification, and support faster and more consistent diagnosis by relying on machine‐based evaluation of the data rather than manually through expert′s examination of the collected data [10].

As a significant subdiscipline of AI, DL trains artificial neural networks using large datasets in such a manner that the relevant patterns can be learned automatically and applied to making decisions. CNNs have become very effective architectures in visual recognition and classification tasks in medical imaging, especially OCT analysis, in particular [11, 12]. CNNs can be used to extract hierarchical image features, starting with simple edges and textures, and moving on to complex pathological structures linked to retinal disease. This automated feature‐learning capability can be of great help in OCT since even highly‐trained clinicians can fail to detect tiny differences in the retinal layers.

These subtle effects are more so in the early stages of the disease or in subtle structural changes associated with the diseases like AMD and diabetic retinopathy (DR). CNN‐based systems, when trained on large collections of OCT images, are more consistently and efficiently able to identify these complex visual patterns than purely manual examination, and also maintain reproducibility across repeated evaluations and reduce diagnostic workload in large‐scale screening programs in everyday clinical practice and referral services.

DL has demonstrated significant value on OCT‐related tasks like retinal layer segmentation, disease classification, abnormality detection, and image‐quality improvement [13]. CNN models are particularly applicable to automatic retinal layer segmentation, which is vital in such disorders as glaucoma and DME, where accurate measurement of retinal thickness is essential for diagnosis [14, 15]. After the segmentation, DL algorithms can categorize OCT scans and identify diseases, including CNV, drusen, or abnormal vascular formations [16]. DL also helps in detecting subtle abnormalities, such as retinal nerve fiber layer thinning in glaucoma or early AMD‐related changes, thus making it possible to diagnose and intervene clinically earlier. Furthermore, the use of generative adversarial networks (GANs) to improve the quality of acquired OCT scans, via noise suppression and image refinement, is increasing to enhance segmentation and disease detection when the acquired images are degraded. The functions are indicative of the increasing role of DL in automated retinal disease evaluation and enhanced patient care.

There are several benefits of DL to OCT‐based detection and diagnosis of retinal diseases. One key advantage is that it can be automatic and has a high processing speed which allows trained models to process OCT images much faster than human observers—a benefit that is useful in busy clinical environments that require rapid decision‐making. DL models, especially those trained using large and diverse datasets, can be very accurate and precise, reducing the risk of human error and helping to ensure that subtle disease markers, such as signs related to DR, are not overlooked. Such systems have even equaled or even exceeded expert performance in particular applications. Moreover, the DL frameworks can be scaled and can be applied in a wide variety of healthcare settings, including tertiary hospitals and smaller clinics and telemedicine platforms, where ophthalmology knowledge is insufficient. Such models can also help in personalized management by identifying retinal diseases and estimating their severity, to help clinicians design patient‐specific treatment plans. To take an example, the early detection of AMD can result in timely treatment and potentially avert the progressive loss of visual function. Moreover, OCT‐based DL can be integrated with complementary imaging modalities, such as fundus photography [17, 18] and fluorescein angiography [19, 20] and help expand the scope of diagnostic assessment, refine the treatment plan, and improve patient outcomes.

### 1.2. Motivation

Retinal diseases, including DME, CNV, AMD, and glaucoma remain a cause of impaired vision and blindness in the world today [7]. Their increased prevalence exerts pressure on the healthcare services, as the good management is linked to the early diagnosis and timely therapeutic intervention. OCT has emerged as a method of ophthalmic imaging, which has created noninvasive, high‐resolution cross‐sectional images of retinal anatomy. However, despite the fact that OCT offers a significant amount of diagnostic data, its interpretation is difficult and requires trained ophthalmic specialists in its interpretation of the data [9]. This means that regular assessment may be time‐consuming, influenced by interobserver variations and hard to carry out on a large scale where the resources of the experts are scarce.

To address these issues, automated retinal image interpretation has become relevant as a field of research. AI and DL have proven to be quite capable in improving OCT analysis through automatic learning of features, disease detection, abnormality detection, and assistance in clinical decision‐making [10]. In particular, CNN‐based architectures have demonstrated high potential to learn multilevel representations of OCT scans, and to recognize subtle retinal structural changes that may not be identified consistently during manual inspection [11, 12]. Nevertheless, a number of DL systems still demand large annotated datasets and deep computational resources, which can limit their practical use in the medical field.

EfficientNet provides an appropriate solution because it balances well between the classification accuracy and computational requirement. Its scaling mechanism of representation learning requires fewer parameters, and therefore it is suitable to medical image analysis in limited‐resource settings. Furthermore, transfer learning facilitates EfficientNet to reuse the knowledge gained with large‐scale reference datasets and apply it to retinal OCT classification, thus eliminating the need to have huge volumes of expert‐labeled data. This benefit is especially helpful in the field of ophthalmology, where annotation by experts is expensive, labor‐intensive, and time‐consuming.

For to these reasons, this research paper is aimed at designing an automated OCT‐based system to classify retinal diseases that is both high in accuracy and computationally feasible. The proposed method, which incorporates the transfer‐learned EfficientNet features with a SVM classifier, is expected to enhance the recognition of retinal classes such as DME, CNV, and drusen, as well as improve stability in low‐data conditions. In general, the work is designed to support ophthalmologists by developing a reliable decision‐support system that is able to accelerate the diagnosis, decrease variability, and enhance access to quality retinal screening in underserved healthcare settings.

## 2. Literature Review

### 2.1. Development of EfficientNet

The previous convolutional DL networks, such as VGG, ResNet, and inception have demonstrated impressive performance in image classification tasks. They frequently demand significant computational resources because of their large number of parameters, deeper structures, and high memory demands. Their practicality in medical imaging systems can be limited by such constraints in practice, which reduce their reliance on hardware and enhance their deployment of models. To address these issues, EfficientNet was introduced as a current CNN architecture that aims to achieve better classification performance at a constant computational cost. The most important contribution of EfficientNet is its scaling mechanism of compounds, which is through this mechanism that the network depth, width, and input image resolution are scaled up in a balanced manner. In contrast to other traditional scaling techniques that tend to grow only one aspect of the model, EfficientNet grows all three dimensions simultaneously in a systematic approach. This enables the network to gain a higher feature representation with fewer parameters and relatively lower cost of computation.

The main concept of EfficientNet is to ensure a reasonable balance between the capacity to learn and the efficiency of the resources. Through scaling the architecture in a coordinated manner, EfficientNet can enhance the predictive performance without generating unnecessary computational load. The design has allowed EfficientNet models to deliver highly competitive classification results in a wide range of image recognition tasks with fewer resources than a number of traditional DL architectures. Such benefits render EfficientNet extremely applicable to medical image analysis, where the accuracy of the diagnosis should be balanced with practicability. Lightweight and accurate models are much needed in clinical applications,

particularly when there is a large image dataset, or when the computing infrastructure is limited. EfficientNet comes in a variety of versions, B0–B7, with each version having a different degree of complexity and representational capability. EfficientNet‐B0 is the smallest variant and offers high feature‐learning capability but it also needs more computer power.

### 2.2. EfficientNet to Interpret Medical Images

The performance of EfficientNet in providing high‐quality classification with less parameter complexity has prompted its application in medical image analysis, especially in retinal disease detection using OCT [21]. EfficientNet has significant advantages in ophthalmology since it is characterized by both good diagnostic precision and reduced computational loads. It is more particularly useful in smaller hospitals, remote medical facilities, telemedicine services, and low‐resource clinical environments, where more advanced hardware might not be readily available to support heavier networks like VGG or ResNet. EfficientNet has been used in OCT‐based studies to identify retinal diseases/conditions such as DME, CNV, and AMD. As these diseases can lead to irreversible vision impairment, once the disease is diagnosed, automated, and accurate image analysis in OCT has significant clinical implications.

Besides disease classification, EfficientNet has also been explored in other applications in ophthalmic image‐processing, including retinal layer segmentation and localization of pathological changes. They consist of clinically relevant manifestations, including fluid areas, retinal thickening, and abnormal vascular formations, which are significant to assess the presence and severity of the disease. These applications demonstrate that EfficientNet is not restricted to image‐level prediction but can be used to provide larger scale diagnostic evaluation in retinal imaging. More recent studies have examined hybrid models consisting of EfficientNet and traditional machine learning classifiers, especially SVMs. EfficientNet is usually employed in these frameworks to extract deep visual features on OCT images and the final categorization is done by the SVM classifier based on the extracted feature space. This combination is applicable in those cases when the available dataset is limited, since deep feature extraction can identify meaningful patterns of images, whereas margin‐based SVM classification can enhance class separation and generalization. Hence, the new hybrid systems that can be created based on EfficientNet offer a promising foundation upon which to develop accurate, efficient, and clinically applicable retinal disease classification models.

### 2.3. Transfer Learning With EfficientNet

One of the major difficulties in medical image analysis is the shortage of large and accurately annotated datasets. Unlike general image datasets, medical datasets usually require expert labeling by trained clinicians or specialists. This process is expensive, time‐consuming, and dependent on professional knowledge. In retinal imaging, accurate labeling of OCT scans may require ophthalmologists to identify disease‐specific patterns and structural abnormalities. Because of these challenges, many medical imaging studies are conducted using relatively small datasets compared with general computer vision research. Transfer learning has become an effective solution to this limitation. Instead of training a DL model completely from the beginning, transfer learning uses a model that has already learned useful visual representations from a large dataset such as ImageNet. These previously learned features can then be adapted to a new medical imaging task. EfficientNet is well suited for this approach because its architecture provides strong feature‐learning ability with efficient parameter usage. For OCT image classification, transfer learning allows EfficientNet to reuse general visual knowledge and adjust it toward recognizing retinal disease patterns [11, 12]. Through transfer learning, EfficientNet can learn to identify subtle disease‐related abnormalities in OCT images, even when only a limited number of training samples are available. These abnormalities may include intraretinal fluid, subretinal fluid, drusen deposits, retinal thickening, and irregular vascular structures. Such features are often important indicators of retinal disease. By fine‐tuning a pretrained EfficientNet model, the system can improve its ability to detect these clinically meaningful patterns without requiring an extremely large dataset. The other advantage of transfer learning in image classification is that it reduces the training period and computing cost. The training of a deep model demands a large volume of data, lengthy training time, and huge computing capabilities. However, a pretrained EfficientNet model possesses many valuable low‐level and mid‐level visual representations such as edges, texture, shape, and object representation. Such learned representations can then be tuned for OCT images, thus reducing training time and computing capability. Such an approach is highly advantageous for clinical environments where there might not be enough data and computing resources available for a new project. EfficientNet‐based transfer learning has proven effective in various fields of medical imaging. For instance, it has been used to classify DR, medulloblastoma tumors, breast cancers, COVID‐19 cases using chest x‐rays, MRI brain tumors, skin lesions, colon cancers, and diabetic foot diseases [22, 23]. Many of these tasks have demonstrated high classification accuracy, stability, and computing efficiency when compared with a number of traditional CNNs. In summary, EfficientNet is an ideal structure for medical image classification because of its scalability, balance between model expansion, and suitability for transfer learning. The feature to produce consistent results with relatively fewer parameters makes it perfect for OCT‐based classification of retinal diseases. The use of EfficientNet within hybrid machine learning techniques such as SVM increases its effectiveness in medical environments where labeled data and computational capabilities are scarce.

## 3. Proposed Methodology

This paper proposes a DTE‐SVM hybrid framework, which stands for Deep Transferred EfficientNet and Support Vector Machine, to develop an automated multiclass classification system for OCT images of retinal diseases. The proposed technique consists of a deep transfer‐learned EfficientNet‐based backbone to produce deep image features followed by a SVM classifier. EfficientNet will be used as the deep feature extraction stage, whereas SVM will conduct the final classification of disease categories based on the extracted deep image features. The rationale behind such a hybrid structure is to enhance discrimination between similar‐looking retinal diseases in OCT scans. It is known that retinal disorders may have certain common structural characteristics, which make automatic classification difficult. EfficientNet will help identify relevant structural features, whereas SVM will be used to build an optimal discriminating hyperplane between the different classes. The proposed framework intends to improve robustness and efficiency of classification and provide stable performances. The overall methodology includes multiple steps, namely, organization of the OCT data according to disease categories, data stratification, preprocessing of the OCT images, adaptation of transfer‐learned EfficientNet models, generation of deep features from the OCT images, SVM classification, and evaluation. After the dataset preparation step, efficient stratification is conducted. Then, the OCT images go through preprocessing procedures. Then, EfficientNet models are adapted to learn specific OCT image patterns. The next step is the feature generation stage where deep features are created from OCT scans. Finally, SVM takes generated features as inputs and classifies them into different disease categories. The novelty of this research is associated with three particular features. First, EfficientNet and SVM models are integrated into a hybrid framework to conduct automatic classification of retinal diseases in OCT images. This combination allows leveraging the advantages of both approaches and combining deep features with SVM‐based decision modeling. Secondly, the study compares several EfficientNet variants (B0, B1, B2, B3, and B4) in one hybrid framework to find out how they affect the classification process. All five networks were used under the same conditions; thus, comparisons are more valid than those made under various settings. Thirdly, this paper evaluates the potential of lightweight EfficientNet models over deeper ones to see if more layers result in better classification accuracy in OCT images. This part is crucial since bigger networks do not always perform better and can be computationally costly. Therefore, the proposed DTE‐SVM framework is expected to offer an efficient approach to OCT classification.

### 3.1. Description of the Dataset

To evaluate the proposed DTE‐SVM framework, a carefully selected working subset of 8767 OCT scans from the main, well‐known OCT2017 database is used for the experimental assessment. These images are divided into four clinical diagnosis groups: CNV, DME, drusen, and normal controls. The first three indicate specific pathological retinal abnormalities, characterized by specific structural features, including fluid accumulation, tissue structure anomalies, retinal deposits, or abnormal vascular changes, whereas the normal category is a healthy control tissue array. This means that the working subset is a well‐balanced mix of pathological and healthy retinal scans, which offers a mathematically well‐rounded data setting that can be challenging and provide a good test of how well‐automated ophthalmic screening tools perform on datasets that are not all images of a single type.

The full repository includes over 84,495 images, but running a full 10‐fold cross validation across five different deep architectures (EfficientNet B0–B4) would be very computationally intensive with a very high memory usage, as noted in [5]. To overcome this practical constraint and reduce operational expenses, a representative, stratified subset comprising exactly 10.37% (rounded to 10.4%) of the primary database was taken out. Instead of using random sampling that can introduce sampling bias and affect the class distribution (standard and naive random sampling), a strict class‐wise stratified sampling strategy was used. For each case, this partitioning was witnessed to be as follows: 2200 CNV, 2190 DME, 2189 drusen, and 2188 normal [5]. This exact setup ensures that all the retinal patterns are represented correctly, and that the underlying localized class distributions and pathological variations of the parent database are preserved.

By selecting a subset that is structurally identical, but smaller than the original, the pipeline can be comprehensively tested for robustness, generalization, and its applicability in real‐world clinical settings with limited time, hardware resources, and computer power. With this smaller and representative data profile, we successfully test our hybrid DTE‐SVM pipeline and validate its high data efficiency and structural stability as shown in Table 1. This not only provides stable clinical‐level diagnostic accuracy but also drastically reduces data‐ingestion speeds and hardware demands, making it a highly practical solution for integration deployed within a scalable clinical decision‐support and telemedicine network.

**Table 1:** Overall and class‐wise performance comparison of DTE‐SVM‐B0 to DTE‐SVM‐B4 over all folds.

Model	Precision	Recall	F1‐score	Accuracy	Std. dev.	CNV F1	DME F1	Drusen F1	Normal F1
DTE‐SVM‐B0	**0.9074**	**0.9076**	**0.9073**	**0.9076**	0.0103	**0.9631**	**0.9165**	**0.8724**	**0.8771**
DTE‐SVM‐B1	0.8916	0.8915	0.8910	0.8915	0.0139	0.9495	0.9007	0.8579	0.8560
DTE‐SVM‐B2	0.8977	0.8978	0.8974	0.8978	**0.0066**	0.9559	0.9122	0.8571	0.8644
DTE‐SVM‐B3	0.8929	0.8929	0.8925	0.8929	0.0095	0.9486	0.8938	0.8589	0.8688
DTE‐SVM‐B4	0.8947	0.8947	0.8944	0.8947	0.0069	0.9585	0.8943	0.8590	0.8656

*Note:* The bolded values indicate the best performance achieved for each evaluation metric.

### 3.2. Training, Validation, and Test Splits of the DTE Stage

When building an end‐to‐end DTE stage, the dataset was further split into three parts: training, validation, and test splits, following the stratified splitting approach according to the ratio of 70%–15%–15%. In other words, 70% of OCT images per diagnostic group were allocated to the training split, 15% of them to the validation split, and the remaining 15% to the test split.

For each diagnostic group *c*, one could express the data split in terms of

Nctrain=0.70NcNcval=0.15NcNctest=0.15Nc..

As splitting was implemented separately for each class, it ensured a balanced distribution of the images across three subsets regardless of whether it is about CNV, DME, drusen, or normal images.

In turn, the training subset was employed to perform fine‐tuning of the pretrained model and enable it to learn OCT retina‐related features. Meanwhile, the validation subset was used to monitor learning progress, check convergence, adjust the learning rate, and implement the early stopping technique.

It should also be noted that the test subset was not involved in any of the above steps since its goal was to conduct experiments on unseen data. Only after model optimization was it employed for evaluation purposes. Thus, such approach to performing experiments yielded a more objective assessment of the proposed DTE stage.

### 3.3. Image Preprocessing and Standardization Pipeline

A unified image‐processing algorithm has been strictly followed prior to feature extraction, in order to guarantee absolute algorithmic reproducibility. The raw ophthalmic OCT scans may naturally have different raw aspect ratios and pixel densities, so these are dynamically resized to a uniform spatial resolution of 224 × 224 pixels across three parallel color channels, which ensures the architecture constraints of the EfficientNet input layers [24]. The matrices are duplicated across three uniform parallel channels to meet the desired matrix dimensions of the pretrained networks for the color channel dimensions.

Once the spatial resizing is done, the intensity normalization is done by feeding the tensor arrays to the model‐specific preprocess_input utility. This mathematically transforms the raw 8‐bit integer pixel intensities (spanning [0, 255]) into normalized floating‐point vectors in strict range [–1, 1].

Data shuffling and online data augmentation methods (such as shearing, artificial scaling of the data) were purposefully turned off (shuffle = false) during the primary DTE‐SVM feature‐mining loop. This ensures the data are ingested in a consistent order, so there is no risk of mismatching the order of the deep feature vectors and the categorical labels for training purposes. This rigid configuration ensures that performance variations are strictly a function of the feature space and maximum‐margin hyperplane boundaries, rather than artifacts of synthetic data expansion.

### 3.4. Hyperparameter Framework and Optimization Suite

There is a hyperparameter framework and optimization suite available. The parameters of the deep feature extraction and support vector classification steps have been carefully standardized in order to ensure absolute reproducibility and architectural transparency of our hybrid pipeline:

• Raw input OCT scans are passed to the pipeline, which processes them to achieve a uniform spatial resolution of 224 × 224 × 3 pixels to align with the spatial constraints of the network.
• The deep convolutional backbone is set up using the EfficientNet family of variants (variant‐wise from EfficientNet‐B0 to EfficientNet‐B4), which are pretrained on ImageNet using large‐scale natural data and hence learn strong and hierarchical features of visual data.
• To take advantage of the deep network only as a local feature extractor and avoid the end‐to‐end conditional probability network training, the standard fully connected Softmax top layers are removed for 1000 classes with the command include_top = false given to Keras [5].

The Global Average Pooling 2D (GAP2D) layer is appended directly after the frozen convolutional base to collapse the spatial dimensions of the remaining activations. It computes the spatial mean along the tensor dimensions and yields highly informative, compact, and low‐dimensional deep feature representations for the last layer outputs, successfully mapping them.

• The size of the resulting deep feature vector dimensions is as follows: 1280 dimensions for EfficientNet‐B0 and EfficientNet‐B1 backbone, 1408 dimensions for EfficientNet‐B2, 1536 dimensions for EfficientNet‐B3, and 1792 dimensions for EfficientNet‐B4 variant.
• Batch allocation strategy: To keep the gradient calculation consistent and allocate equal and constant memory on the hardware, images are ingested in fixed minibatches of 32 images per operational step.

The feature elements extracted using the feature extraction routine are normalized using a *Z*‐score standardization routine via a structured pipeline StandardScaler [24] before being classified. This transformation is known as centering and standardization and centers the data around the mean value (*μ* = 0) and standardizes it to a unit variance (*σ* = 1). This step is vital since it prevents the features that have a wide numerical range from dominating the smaller but very discriminative ones in the next hyperplane optimization step. The feature sets are then standardized and directly fed into a linear support vector classifier (SVC) for structural optimization [24].

The support vector decision boundaries are realized on the basis of applying a strict linear kernel function (**w** ^*T*^ **x** + *b*). This secondary machine learning classification step is detailed below:

• The soft‐margin regularization penalty parameter (*C*) is fixed at a very high value of 10 [8]. This results in the optimization suite being severely penalized for misclassifications when training and sets a strict margin to ensure the greatest geometric separation between neighboring retinal disease margins in the high dimensional feature space.
• The internal probability estimator can be used to output continuous confidence intervals in addition to hard class predictions using Platt scaling (probability = true). This is a subroutine that inserts a logistic regression model directly into the distance‐to‐hyperplane measure obtained by an SVM.
• The whole hybrid framework is extensively validated using a 10‐fold cross validation method with a deterministic and a stratified approach. This configuration ensures that original categorical class ratios are kept 100% balanced and unbiased by evaluation throughout the 10 validation cycles [22].

### 3.5. Transfer Learning and Feature Extraction Using EfficientNet

In order to adapt EfficientNet architectures for classification of retinal images with OCT imaging, we used a two‐step transfer‐learning algorithm. At the first step, the pretrained weights of the convolutional backbone of the EfficientNet model trained on ImageNet were used without updates. That is, during this step, only newly created classification layers were trained. Thus, the model was able to start adapting to the OCT medical data while keeping general visual representations obtained through learning from extensive image data. Pretrained models learn visual representations at different abstraction levels, from basic elements, such as edges, textures, contours, shapes, structures, and even semantic information that helps to learn representations of medical imagery.

At the second step, higher layers of the EfficientNet convolutional backbone were unlocked to perform fine‐tuning. The purpose of fine‐tuning at this stage was to adjust internal visual representations of the network toward the OCT problem while avoiding drastic and unstable changes to learned parameters. As a result, the adapted EfficientNet became more capable of recognizing clinical OCT patterns, such as abnormalities in retinal layers, fluid accumulation, tissue deformation, and deposits. Transfer learning was used to improve the capability of extracting disease‐specific features using OCT scans, especially when the number of labeled medical images was small. Training a deep neural network from scratch requires an extremely large dataset and lots of computing power. On the contrary, transfer learning makes it possible to reuse pre‐existing visual representations and adjust them to the task of medical image processing.

Even though the end‐to‐end DTE architecture was a part of the methodological framework considered in this study, our main focus is the hybrid DTE‐SVM classification scheme. In this case, the EfficientNet model is used primarily as a feature extractor, whereas the SVM plays a role in classifying input medical images.

Let us denote by *x* *i* the *i*‐th OCT image from the dataset. Then after transfer learning, the EfficientNet convolutional backbone transforms the input image to a high‐level deep feature vector as follows:

(1) zi=φθxi,

where *φ*_*θ*_(·) is a feature extraction function of the EfficientNet model after the transfer learning process, and *z*_*i*_ is the resulting deep feature representation of input image *x*_*i*_. This representation contains discriminative information about the OCT image, which is then passed to the SVM classifier.

### 3.6. Hybrid DTE‐SVM Classification

A hybrid classification scheme was chosen for the framework, rather than utilizing the softmax output of the CNN as the final classifier. Specifically, in the proposed method, deep feature vectors were first extracted using the transfer‐learned EfficientNet model, which were then classified into one of several classes by SVM.

The key motivation behind the use of the proposed hybrid approach is to increase the separation among the retinal disease categories that are hard to distinguish visually based on OCT images. Different retinal diseases can have very similar image features and only slight distinctions between them. EfficientNet has demonstrated outstanding abilities to learn hierarchical visual features of OCT images, whereas SVM is good at drawing decision boundaries. Therefore, combining these approaches allows improving the discrimination power of the classification system.

In DTE‐SVM approach, EfficientNet is responsible for extracting features from the OCT images, whereas the SVM acts as the decision component of the framework. Deep features obtained through EfficientNet provide rich information about retinal image structure, and SVM leverages such representation to properly classify the input images into their respective classes. This makes DTE‐SVM a suitable approach to solve the medical image classification task, where precise class separation is crucial for the classification performance.

For each sample image *x*_*i*_, the classification result can be calculated with the help of decision function:

(2) y∧i=argmaxk wkTzi+bk,

where ${\overset{\wedge }{y}}_{i}$ represents the predicted class of image *x*_*i*_, *z*_*i*_ is a vector of deep features for sample image, *w*_*k*_ and *b*_*k*_ represent weight vector and the bias term correspondingly for each class *k*.

The deep features obtained with the help of EfficientNet were standardized before classification with the help of SVM classifier. Standardization of the features made them lie within a common numerical range, making the training process of SVM more stable and improving class separation between the retinal diseases.

### 3.7. Stratified 10‐Fold Cross‐Validation for the Main DTE‐SVM Evaluation

The main performance evaluation of the proposed hybrid DTE‐SVM framework was carried out using stratified 10‐fold cross‐validation. This evaluation method was selected to obtain a more stable and reliable measurement of model performance across the complete working dataset. Instead of depending on a single train‐test split, the dataset was divided into 10 separate folds, and the classification experiment was repeated across different training and evaluation combinations.

The working dataset contained 8767 OCT images. During the stratified 10‐fold cross‐validation process, these images were divided into 10 nearly equal groups while preserving the class distribution in each fold. This means that every fold contained representative samples from all four diagnostic categories: CNV, DME, drusen, and normal. Maintaining this class distribution was important to ensure that no retinal disease category was overrepresented or underrepresented during evaluation. In each cross‐validation iteration, nine folds were used for training the DTE‐SVM framework, whereas the remaining fold was used for evaluation. This process was repeated 10 times, and each fold was used once as the evaluation set. As a result, every OCT image in the working dataset contributed to testing exactly once and was also used for training in the remaining iterations.

In each run, approximately 7890 images were used for training, whereas nearly 877 images were used for evaluation.

The fold formation was performed in a class‐wise manner. For each class *c*, the available OCT images were divided into 10 stratified parts, so that each fold contained approximately

Nc10,

samples from class *c*. Therefore, during each cross‐validation run, the training portion for class *c* contained approximately

9Nc10,

while the held‐out evaluation portion contained approximately

Nc10.

This stratified design ensured that all classes were fairly represented in every training and evaluation cycle. Such a procedure is especially important in medical image analysis because an imbalanced evaluation split can produce misleading performance results. If one disease class is poorly represented in a fold, the model evaluation may not accurately reflect its ability to classify that condition. By preserving the class distribution across all folds, the cross‐validation process provided a more dependable assessment of the proposed framework.

The final results reported in this work were obtained from the stratified 10‐fold cross‐validation of the DTE‐SVM framework using the complete 8767‐image working dataset. This evaluation served as the primary experimental protocol of the study. The separate end‐to‐end DTE split was included only to complete the methodological workflow, whereas the main performance analysis was based on the cross‐validated hybrid DTE‐SVM model.

### 3.8. Training Configuration

During the process of training in the DTE stage, the EfficientNet models were optimized using the Adam optimization technique with a categorical cross‐entropy loss function. Optimization of the model parameters was done through minibatch gradient descent by updating the model parameters in batches during the processing of the OCT images. To minimize the chances of overfitting in the optimization process, the early stopping technique was used during the training phase. In addition, the learning rate of the model was dynamically adjusted using a schedule.

The above training parameters were consistently maintained in all the EfficientNet variations ranging from EfficientNet‐B0 to EfficientNet‐B4. The use of identical training parameters ensured that all the EfficientNet variations underwent fair and consistent comparison. Since the optimization technique, loss function, training technique, and regularization techniques were kept identical for all variations, any observed differences in their performance would be because of variations in their architecture.

In the DTE‐SVM stage, feature vectors were obtained using the fold partitions based on the classes as outlined in the previous section. Standardization of the feature vector data was done before feeding them into the SVM classifier. Feature standardization ensured that the features were on a comparable scale, which improved the stability of the SVM learning and ensured clear separability between diagnostic categories.

The framework was evaluated on different criteria such as accuracy, precision, recall, F1 score, sensitivity, specificity, receiver operating characteristic (ROC), and confusion matrix analysis. The above criteria were selected to ensure a thorough assessment of the model behavior from an overall and class perspective. The latter is necessary since it is crucial to analyze not only the general accuracy but also the reliability of disease identification of each diagnostic category.

### 3.9. Workflow Summary

The entire process of the proposed retinal diseases classification algorithm is as follows:

1. Images were taken and sorted into four diagnostic classes including CNV, DME, drusen, and normal.
2. Class‐based stratified data organization method was applied in order to maintain the balance between diagnostic classes.
3. Preprocessing and augmentation of images were done to preprocess OCT2017 images for training and improve image variability.
4. EfficientNet‐B0, EfficientNet‐B1, EfficientNet‐B2, EfficientNet‐B3, and EfficientNet‐B4 were initialized with ImageNet weights.
5. A two‐stage transfer learning technique was used for adapting EfficientNet models to OCT2017 images.
6. Deep retinal features were extracted from transfer‐learned models based on EfficientNet backbones.
7. Feature extraction results were standardized in order to make numeric values uniform.
8. SVM classifiers were developed and tested using standardization of EfficientNet feature vectors.
9. Stratified 10‐fold cross validation was executed on the entire 8767 images dataset.
10. Finally, the performance of the model was calculated through different types of evaluation techniques.

### 3.10. Methodological Rationale

The proposed methodology was developed to overcome important challenges commonly encountered in OCT‐based retinal disease classification. These challenges include the limited availability of expert‐labeled medical images, the need for computationally efficient diagnostic models, and the difficulty of distinguishing retinal diseases that may show visually similar patterns in OCT scans. Since medical image datasets are often smaller than general computer vision datasets, transfer learning was used to make effective use of pretrained EfficientNet representations and reduce the need for training a deep model from the beginning.

The DTE phase helps to make the learned features more suitable to the OCT problem. Transfer adaptation leads to the network being able to detect useful patterns that correspond to the appearance of structural variations associated with the retinal image. In addition to the application of the traditional CNN classification layer, a SVM classifier was used in order to provide additional support for the decision‐making process through margin‐based decision boundaries. The key

difference of this methodology from standard procedures lies in the class‐wise stratified data splitting technique. This is crucial in case of medical image analysis since imbalanced dataset structure could be responsible for unreliable performance metrics due to possible bias. Stratified 10‐fold cross‐validation was employed for this task in parallel with stratified splitting for a more stable and reliable evaluation. This approach helped to improve the repeatability of the evaluation since the performance results obtained on one random data splitting would not be representative.

As shown above, the proposed DTE‐SVM framework provides a well‐balanced way of automated retinal disease classification from OCT images. Due to integration of transfer learning, class‐preserving data handling, standardized deep feature extraction, and SVM classification techniques, it should help perform reliable multiclass retinal image analysis.

## 4. Results and Discussion

### 4.1. Description of the Experimental Setup

The experiments were conducted to assess the effectiveness of the proposed DTE‐SVM variants, namely DTE‐SVM‐B0, DTE‐SVM‐B1, DTE‐SVM‐B2, DTE‐SVM‐B3, and DTE‐SVM‐B4, for automated retinal disease classification from OCT scans. The working dataset employed in this study contained 8767 OCT images distributed across four clinically relevant categories: CNV, DME, drusen, and normal. To obtain reliable and statistically meaningful estimates, stratified 10‐fold cross‐validation was adopted so that the class distribution remained preserved in each fold. In every split, nearly 7890 samples were used for training and 877 samples were used for evaluation. All model variants were trained under identical settings using the Adam optimizer and categorical cross‐entropy loss. Early stopping and checkpointing were used to minimize overfitting and to retain the best performing configuration. Each backbone was trained for a maximum of 10 epochs, thereby ensuring a fair comparison across all EfficientNet variants.

### 4.2. Average Classification Metrics Across All Folds

In Table 1, we provide the performance analysis of DTE‐SVM‐B0 to DTE‐SVM‐B4 for all folds. From the above table, we can see that DTE‐SVM‐B0 produces the best precision, recall, F1‐score, and accuracy scores, and therefore DTE‐SVM‐B0 is considered the best model overall when evaluated using classification metrics. On the other hand, DTE‐SVM‐B2 is observed to have the smallest standard deviation and is thus the most stable across folds. When it comes to the individual classes, CNV is found to have the best F1‐scores in all the backbones used, whereas drusen is the toughest class to classify.

### 4.3. Comparison With Representative Baseline Models

To determine whether the proposed DTE‐SVM framework improves upon standard CNN‐based transfer learning classifiers, additional baseline experiments were conducted using ResNet50, DenseNet121, and EfficientNetB0 with a softmax classification head. Table 2 summarizes the comparison. The proposed DTE‐SVM‐B0 achieved the highest accuracy, precision, recall, and F1‐score among all tested models. Specifically, it improved accuracy by 4.36 percentage points over ResNet50, 3.37 percentage points over DenseNet121, and 1.70 percentage points over EfficientNetB0 softmax. These results demonstrate that the improvement is not due only to the EfficientNet backbone, but also to the hybrid integration of deep feature extraction and SVM‐based decision boundaries.

**Table 2:** Comparison of representative transfer learning baselines with the proposed DTE‐SVM‐B0 framework.

Model	Accuracy	Precision	Recall	F1‐score
ResNet50	0.8640	0.8756	0.8640	0.8632
DenseNet121	0.8739	0.8913	0.8739	0.8745
EfficientNetB0 softmax	0.8906	0.8915	0.8906	0.8889
**DTE-SVM-B0**	**0.9076**	**0.9074**	**0.9076**	**0.9073**

*Note:* The bolded values indicate the best performance achieved for each evaluation metric.

### 4.4. DTE‐Only Performance and Ablation Analysis

To explicitly evaluate the contribution of the SVM classifier, the end‐to‐end DTE models using softmax classification were compared with their corresponding DTE‐SVM variants. Table 3 reports the DTE‐only results. Among the DTE‐only models, DTE‐B3 achieved the best performance, with an accuracy of 0.8906 and F1‐score of 0.8902. However, all DTE‐only models remained below the best DTE‐SVM configuration, confirming that the SVM classifier improves the final decision stage.

**Table 3:** Performance of DTE‐only EfficientNet models with softmax classification.

Model	Accuracy	Precision	Recall	F1‐score
DTE‐B0	0.8822	0.8860	0.8822	0.8825
DTE‐B1	0.8891	0.8907	0.8891	0.8893
DTE‐B2	0.8708	0.8804	0.8708	0.8709
**DTE-B3**	**0.8906**	**0.8910**	**0.8906**	**0.8902**
DTE‐B4	0.8761	0.8817	0.8761	0.8770

*Note:* The bolded values indicate the best performance achieved for each evaluation metric.

Table 4 presents the direct ablation comparison between DTE and DTE‐SVM. The results show that DTE‐SVM improves performance across all EfficientNet backbones. The largest accuracy gain is observed for B2, where accuracy increases from 0.8708 to 0.8978, corresponding to a gain of 2.70 percentage points. For B0, accuracy increases from 0.8822 to 0.9076, corresponding to a gain of 2.54 percentage points. These consistent improvements confirm that SVM strengthens class separation in the EfficientNet feature space.

**Table 4:** Ablation comparison between DTE and DTE‐SVM across EfficientNet backbones.

Backbone	DTE Acc.	DTE‐SVM Acc.	Acc. gain	DTE F1	DTE‐SVM F1	F1 gain
B0	0.8822	**0.9076**	**+0.0254**	0.8825	**0.9073**	**+0.0248**
B1	0.8891	**0.8915**	**+0.0024**	0.8893	**0.8910**	**+0.0017**
B2	0.8708	**0.8978**	**+0.0270**	0.8709	**0.8974**	**+0.0265**
B3	0.8906	**0.8929**	**+0.0023**	0.8902	**0.8925**	**+0.0023**
B4	0.8761	**0.8947**	**+0.0186**	0.8770	**0.8944**	**+0.0174**

*Note:* The bolded values highlight the improved performance and positive gains achieved by the DTE‐SVM approach compared to the DTE‐only baselines.

### 4.5. Per‐Class Clinical Metrics

Besides global classification measures, per‐class sensitivity, specificity, and accuracy were calculated to offer clinically significant analysis. Sensitivity is the capability of the model to accurately recognize a given retinal class, whereas specificity quantifies how well it can rule out all other classes. Table 5 records these measures of the best performing DTE‐SVM‐B0 model. CNV was found to have the best sensitivity and specificity, and hence its good separability. Drusen and sensitivity in normal was relatively less showing that such categories are still more difficult due to subtlety structural overlap.

**Table 5:** Per‐class clinical metrics of the best‐performing DTE‐SVM‐B0 model.

Class	Sensitivity	Specificity	Accuracy
CNV	0.9736	0.9902	0.9821
DME	0.9220	0.9745	0.9483
Drusen	0.8635	0.9632	0.9314
Normal	0.8713	0.9556	0.9232

### 4.6. Performance Metrics

**Figure 3:**
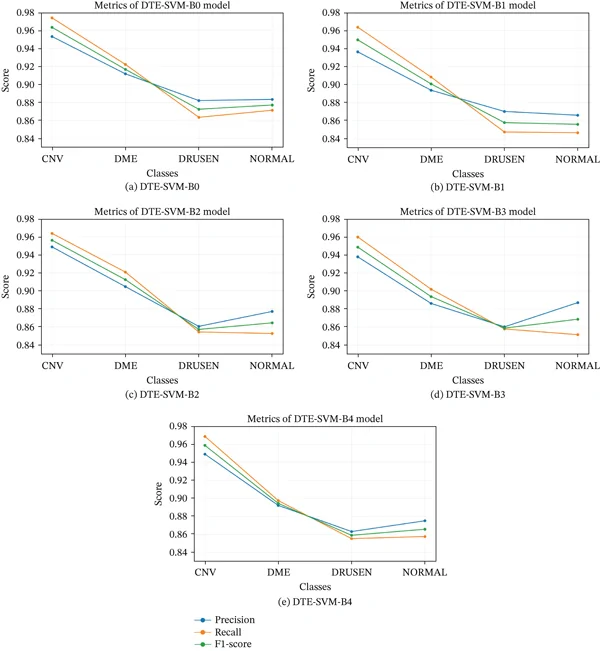
Class‐wise performance metrics of the proposed DTE‐SVM models using EfficientNet backbones from B0 to B4. (a) DTE‐SVM‐B0. (b) DTE‐SVM‐B1. (c) DTE‐SVM‐B2. (d) DTE‐SVM‐B3. (e) DTE‐SVM‐B4.

The proposed DTE‐SVM models were evaluated in terms of four standard measures of classification: accuracy, precision, recall, and. F1‐score. A comparative visual representation of the class‐wise metric behavior of the five variants based on EfficientNet is presented in Figure 3 in CNV, DME, drusen, and normal.

On closer observation of Figure 3, it is evident that there is a consistent pattern in all the models. CNV is the most discriminative type, as indicated by the similarly high scores in terms of classes in all variants. DME also demonstrates a good level of recognition especially in B0 and B2, indicating that the hybrid EfficientNet‐SVM pipeline is able to reproduce the structural retinal features associated with this disease. By contrast, drusen has the lowest class‐wise scores in almost all of the backbones, which means that its retinal patterns are more challenging to differentiate among the rest of the classes. NORMAL has moderate and relatively stable performance, but in general, it is lower than CNV and DME. Combined, the metric profiles assure that the suggested model is very useful with retinal defects that can be easily separated, whereas other less obvious groups remain are more difficult to classify. Among the five variants, DTE‐SVM‐B0 achieved the best mean precision (0.9074), recall (0.9076), F1‐score (0.9073), and precision (0.9076), which proves its high performance in general. DTE‐SVM‐B2 came just behind and as a result of being less, was not as good. Standard deviation proved to be the most consistent. These results suggest that in the proposed DTE‐SVM model, a small and lean backbone may prove more beneficial than more profound options.

### 4.7. Visual Explanations Using ROC Curves

**Figure 4:**
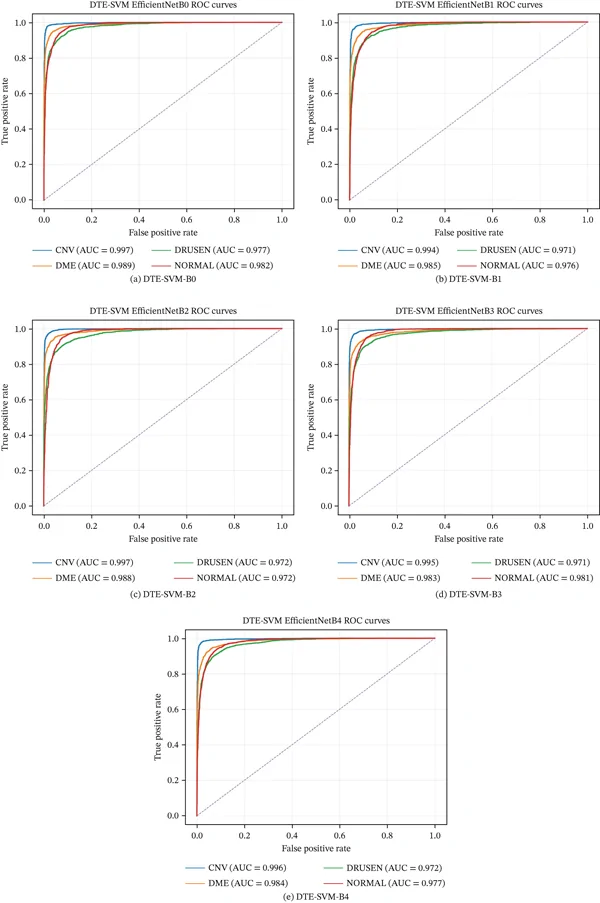
ROC curves of the proposed DTE‐SVM models using EfficientNet backbones from B0 to B4. (a) DTE‐SVM‐B0. (b) DTE‐SVM‐B1. (c) DTE‐SVM‐B2. (d) DTE‐SVM‐B3. (e) DTE‐SVM‐B4.

In order to investigate further the issue of class separability, ROC curves were plotted in all five DTE‐SVM variants. Figure 4 presents a comparative image of the ROC behavior in CNV, DME, drusen, and normal.

The analysis of ROC shows that all models had high‐class discrimination, and the values of the AUC were consistently high across most retinal categories. Meanwhile, the ROC behavior demonstrates that the ranking of the models based on the separability of the classes alone does not necessarily give the same result as ranking them by mean cross‐validation performance. This is particularly important here, since DTE‐SVM‐B0 demonstrated the best average classification scores, DTE‐SVM‐B2, DTE‐SVM‐B3. The highly competitive ROC characteristics of various classes were also exhibited by DTE‐SVM‐B4. Therefore, the ROC analysis is a complement to the folded‐average metrics as opposed to substituting them.

### 4.8. Statistical Results of DTE‐SVM‐B0 Model

According to Figure 4a DTE‐SVM‐B0 exhibited a strong ROC behavior with all four retinal categories. Its curves are kept close to the upper‐left part of the ROC space, which validates high‐class separability and sound discrimination. This observation is completely consistent with Table 1, where B0 achieved the highest overall precision, recall, F1‐score and accuracy. Therefore, B0 is not only powerful in a threshold‐independent sense, but also achieves the best overall performance in terms of classification across folds.

### 4.9. Statistical Results of DTE‐SVM‐B1 Model

The ROC curves of DTE‐SVM‐B1 reveal that this model can be used to achieve competent class separation, especially that of CNV and NORMAL. It performs weaker, however, when compared with the stronger variants; its fold‐averaged performance is weaker. The model records have lower mean precision, recall, F1‐score, and accuracy than B0, and its standard deviation is

the largest of all backbones. These results show that among the variants of B1, it is not as stable and reliable as the other variants in the proposed framework.

### 4.10. Statistical Results of DTE‐SVM‐B2 Model

Figure 4c indicates that DTE‐SVM‐B2 will provide the strongest ROC behavior, especially in the case of CNV and normal. The curves imply high separability of the classes and the profile of B2 in general is highly competitive among the four retinal categories. Moreover, the standard deviation of B2 is the smallest compared with all other variants, so B2 is the most consistent model in the fold‐wise evaluation.

Figure 4 presents a comparative visualization of class separability across the evaluated variants.

### 4.11. Statistical Results of DTE‐SVM‐B3 Model

The ROC behavior of DTE‐SVM‐B3 confirms balanced and strong class separability across all retinal categories. In particular, its curves show that the model learned meaningful retinal representations for DME and CNV. Even so, the aggregate cross‐validation metrics of B3 remain below those of both B0 and B2. This indicates that although B3 is strong from the perspective of ROC‐based separability, it does not deliver the strongest overall classification outcome within the hybrid DTE‐SVM architecture.

### 4.12. Statistical Results of DTE‐SVM‐B4 Model

Figure 4e shows that DTE‐SVM‐B4 also has a good separation of classes, particularly in the case of CNV and drusen. However, even with this very competitive ROC profile, B4 still does not outperform B0 on the fold‐averaged metrics; it is still slightly less good mean accuracy B2 or less. Therefore, it is evident that B4 is effective in class discrimination, but the new results do not support it as the best general model. Rather, it is a competent yet not ideal one among the existing experimental conditions.

### 4.13. Average Confusion Matrix Analysis

**Figure 5:**
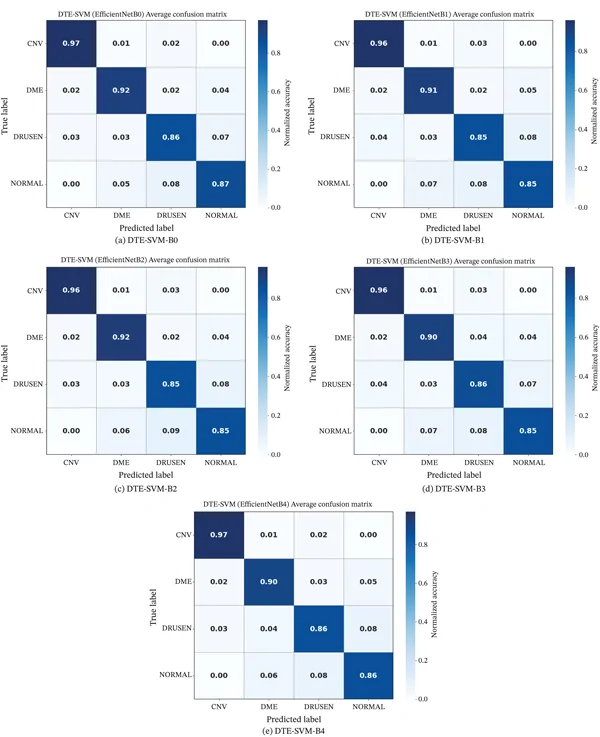
Average confusion matrices over all folds for the proposed DTE‐SVM models using EfficientNet backbones from B0 to B4. The matrices illustrate the class‐wise prediction behavior of each variant across CNV, DME, drusen, and normal. (a) DTE‐SVM‐B0. (b) DTE‐SVM‐B1. (c) DTE‐SVM‐B2. (d) DTE‐SVM‐B3. (e) DTE‐SVM‐B4.

Figure 5 shows the averages of the normalized confusion matrices of the five DTE‐SVM variants. These matrices offer a direct class‐level perspective of prediction behavior in CNV, DME, drusen, and normal.

In all five matrices, the most significant activations are along the main diagonal, which supports the suggestion that the proposed framework has the strongest activations along the main diagonal appropriately categorizes most of the samples in each category of the retina. The most intense diagonal patterns can be noticed in the case of CNV, which is in line with its best class‐wise F1‐scores in Table 1. Comparatively, the off‐diagonal values of drusen and normal are relatively large, which suggests the degree of mutual confusion is relatively high. DME is doing very well across the board; however, even slight spillovers into other categories can be seen. B0 out of the five backbones displays the clearest diagonal dominance in general, with B2 being very competitive and exhibiting an equally reliable structural prediction of class effects.

### 4.14. Evaluation and Results

The new experimental findings indicate that the proposed DTE‐SVM model can be used with high multiclass classification accuracy and with a smaller working subset of 8767 OCT images. This subgroup equates to about 10.4% of the full OCT2017 data and was employed to reduce the cost of training and feature‐extraction, and to maintain the representation classwise. In this reduced‐data environment, DTE‐SVM‐B0 has an average accuracy of 0.9076, precision of 0.9074, recall with a value of 0.9076, and F1‐score of 0.9073, which proves that the proposed hybrid framework can achieve high diagnostic performance without the need to train on full‐dataset. B0 DTE‐SVM was the best overall performer. This finding implies that the lightweight. With an SVM classifier, EfficientNet‐B0 backbone extracts retinal features discriminative enough to support classification tasks. In contrast, more complex models like B3 and B4 did not advantage the performance, indicating that the increase in model depth does not always benefit performance scale up to improved OCT classification in reduced‐data and computationally constrained environments. DTE‐SVM‐B2 achieved the minimum fold‐wise standard deviation, so the most consistent behavior, but still its overall performance was lower than that of B0. According to the results of the classification by classes, CNV is the best categorically classified category with the highest F1‐score and the strongest clinical metrics. DME also exhibits a good level of recognition. Drusen is still the most challenging category that is consistent with its fine patterns of retinal texture and because of its possibly overlapping normal images. The further clinical metric table affirms this tendency, where drusen and normal are less sensitive than CNV and DME. These results highlight the need to further enhance the ability to see the fine patterns of diseases. Comparisons based on the baselines also prove the advantage of the suggested model. DTE‐SVM‐B0 outperformed ResNet50, DenseNet121, and EfficientNetB0 softmax classifiers. The proposed model increased the accuracy by 4.36 percentage points above. Resnet50, 3.37 percentage points over DenseNet121, and 1.70 percentage points over EfficientNetB0 softmax. This confirms that this performance improvement is not merely a result of EfficientNet feature extraction, but also the SVM classifier that is employed in the proposed hybrid framework. The ablation study is yet another

evidence of the effectiveness of the hybrid design. Other models, as compared with DTE only models, are DTE‐SVM was more accurate and had higher F1‐score with all EfficientNet backbones. The greatest growth was in terms of B2, with an increase in accuracy by 2.70 percentage points and an increase in F1‐score by 2.65 percentage points. In the case of B0, the accuracy improved by 2.54 percentage points and F1‐score improved by 2.48 percentage points. Such regular profits indicate that SVM but tightens the separation of classes based on margin in the trained EfficientNet feature space. Overall, the findings prove the most effective combination among all models tested to be DTE‐SVM‐B0. The findings also demonstrate that the light backbones can perform better than the deep variants when used with SVM, such that the proposed method appropriate to computationally efficient OCT‐based retinal disease diagnosis and practical clinical decision‐support applications.

### 4.15. Ablation Studies

The results of the ablation confirm three key observations. To begin with, all variants of EfficientNet turned out to be effective at OCT‐based retinal disease classification, the findings failed to depict an increasing gain with increasing backbone depth. Rather, EfficientNetB0 was the best average classification model and EfficientNet‐B2 demonstrated the most consistent fold‐wise performance. This implies that merely adding more complexity to the model does not necessarily enhance the quality of diagnostics in the proposed DTE‐SVM framework. Second, the findings support the computational superiority of EfficientNet in analyzing medical images. Because EfficientNet when it adopts compound scaling, it is able to give robust feature learning with fewer parameters than most traditional convolutional architectures. It is especially significant in the retinal image analysis, where the models are supposed to sustain both predictive power and computational efficiency to deploy in the real world. Third, the hybrid DTE‐SVM design was more effective as compared with the application of the EfficientNet‐based DTE model alone. Fine‐tuning trained EfficientNet models demonstrated a more robust convergence behavior, and improved discrimination, compared with training with a vanilla network, that is in line with previous transfer learning results in medical image analysis [12, 14, 25, 26]. Besides, it is possible to incorporate an SVM at the last classification step enhanced the strength of the decision‐boundaries on the learned feature‐space, in support of the effectiveness of the hybrid strategy [15, 27]. Overall, these findings of ablation prove that a set of the transfer‐learned EfficientNet features is combined and SVM classification offers a precise and computationally efficient method to recognize retinal diseases using OCT scans.

## 5. Conclusion

This paper has introduced a hybrid DTE‐SVM architecture to automatically classify retinal diseases in multiple classes using OCT images. The suggested model is a hybrid of transfer‐learned. The extraction of EfficientNet features to an SVM decision layer to enhance the ability of the classifier to discriminate and classify its robustness, and its computational efficiency. The experiments were run on a smaller 8767‐image working subset of the OCT2017 dataset, which is corresponding to about 10.4% of the full data set, therefore, saving on the cost of computation, but maintaining representative class‐wise evaluation. Experimental outcomes showed that, DTE‐SVM‐B0 had the best overall performance among those tested. EfficientNet variants, with accuracy of 0.9076, precision of 0.9074, recall of 0.9076, and F1‐score of 0.9073. DTE‐SVMB2 exhibited the least fold‐wise behavior, which suggests that the generalization of the overall behavior is the same across cross‐validation folds class‐wise. It was found that CNV was the most accurately identified and drusen was the most difficult because of delicate visual images and interlude with other retinal types. The baseline comparison has established that the proposed DTE‐SVM‐B0 softmax classifiers outperformed ResNet50, DenseNet121, and EfficientNetB0 softmax classifiers. The ablation experiment also confirmed that the substitution of the softmax classifier by SVM enhanced accuracy and F1‐score on all EfficientNet backbones. These results indicate that the enhancement is not a result of only transfer learning using EfficientNet as well as the more powerful margin‐based decision boundaries brought forth by SVM. In sum, the proposed DTE‐SVM‐B0 framework offers an accurate, robust and computationally efficient solution to OCT‐based retinal disease classification. The results also indicate that more elaborate architectures are not necessarily better and that lightweight backbones are able to provide the best performance when used in conjunction with a suitable classifier. This renders the suggested framework has potential as a practical computer aided ophthalmic diagnosis, especially in a resource constrained clinical environment.

### 5.1. Scope of Future Work

Some promising avenues can be undertaken to continue enhancing the clinical relevance, robustness, and diagnostic ability of the suggested DTE‐SVM framework. A close collaboration with ophthalmologists and retinal specialists to confirm the predictions of the model in the actual clinical practice can be considered as an important next step. Proficient interpretation and input will be helpful to evaluating the practical dependability of the system, redrawing decision lines, and enhancing alignment between algorithmic clinical judgment, and predictions. Further research must also integrate larger and more diverse datasets, such as samples obtained in rural, underserved areas, and demographically diverse populations. This type of expansion would enhance the model applicability to various patient groups and imaging situations, thus facilitating more fair and reliable ophthalmic screening. The systematic optimization of model training and architecture design is another interesting direction of research. Although the present work has shown that DTE‐SVM‐B0 has the best overall classification performance and DTE‐SVMB2 exhibits the most consistent fold‐wise behavior, additional improvements are still possible, though by performing more rigorous hyperparameter tuning. Future research can thus consider other optimization techniques, learning rates, batch sizes, and fine‐tuning policies to further improve accuracy, precision, recall, and F1‐score in all retinal categories. Specifically, it is necessary to enhance the identification of more demanding classes like drusen is still a significant goal. The framework diagnostic scope can be also extended beyond the current classes to embrace other retinal disorders, including AMD, DR, and retinal occlusive diseases, thus making it possible to assess them in a more general way of retinal pathology based on OCT. Another area of enhancement is in multimodal ophthalmic analysis. Combining OCT with complementary structural and vascular data, for example, fundus photography and OCT angiography [28], may offer more detailed information on structure and vascularity, that might enhance early diagnosis of complicated ocular conditions such as retinal vein occlusion (RVO), high myopia, and glaucoma. The construction of a single diagnostic system using a combination of these modalities could significantly enhance sensitivity and specificity as well as enhancing clinical utility. In addition to multimodal expansion, other possible future work directions include lightweight deployment strategies of real‐life screening systems, particularly in resource‐constrained environments where the use of computations is crucial. These developments would assist in the transition of the proposed framework out of the experimental level validation to practical and scalable clinical use.

**Nomenclature**
AMD: age‐related macular degeneration
CNN: convolutional neural network
CNV: choroidal neovascularization
DME: diabetic macular edema
DTE: deep transferred EfficientNet
OCT: optical coherence tomography
ROC: receiver operating characteristic
SVM: support vector machine

## Author Contributions

Siddartha Arekanti: study concept, methodology design, manuscript writing, and careful organization of experimental results. Ch Madhu Bhushan: implementation of the research idea, methodology design, preprocessing, and initial model development. Irfan Alam: reviewed the methodology, provided technical suggestions, supported manuscript revision, and contributed to the final review. Priya Koppuravuri: data preparation, literature review, model tuning, and assistance with the interpretation of analyses. Fazal Shaik: ROC curve analysis, model tuning, performance evaluation, and preparation of result figures and summaries. Md Muzakkir Hussain: supervision of the research work, technical guidance, methodology review, and final manuscript refinement. Firoj Gazi: statistical analysis, cross‐validation, visualization, ablation interpretation, and discussion of future directions.

## Funding

No funding was received for this manuscript.

## Disclosure

All authors have read and approved the final manuscript.

## Conflicts of Interest

The authors declare no conflicts of interest.

## Data Availability

The data that support the findings of this study are openly available in Labeled Optical Coherence Tomography (OCT) for Classification at https://data.mendeley.com/datasets/rscbjbr9sj/1, Reference Number 10.17632/rscbjbr9sj.1.
